# Structural Femoral Shaft Allografts for Anterior Spinal Column Reconstruction in Osteoporotic Spines

**DOI:** 10.1155/2016/8681957

**Published:** 2016-11-22

**Authors:** Bong-Soon Chang, Jong-Hun Jung, Sang-Min Park, Seung Hoo Lee, Choon-Ki Lee, Hyoungmin Kim

**Affiliations:** ^1^Department of Orthopaedic Surgery, Seoul National University College of Medicine, Seoul, Republic of Korea; ^2^Spine Center, Department of Orthopaedic Surgery, CM General Hospital, Seoul, Republic of Korea

## Abstract

This study was to investigate the clinical and radiographical outcomes of anterior spinal column reconstruction using structural femoral shaft allografts in osteoporotic patients. Retrospective analyses of medical records, radiographic parameters, and postoperative complications were performed in twenty-one patients who underwent anterior spinal column reconstruction surgery for osteoporotic vertebral collapse or nonunion. Surgical invasiveness, clinical outcomes, postoperative complications, and radiographic outcomes were evaluated. Ambulatory status and back pain significantly improved. The Cobb's angle of segmental kyphosis significantly improved immediately after surgery with slight progression at the final follow-up. There were two cases of failed reconstruction with marked progression of kyphosis; both were related to loosening of screws rather than subsidence of the graft. Anterior spinal column reconstruction using femoral shaft allografts improved kyphosis and resulted in minimal subsidence and therefore is recommended as an effective treatment option for dealing with osteoporotic vertebral collapse and kyphotic deformity.

## 1. Introduction

Osteoporosis is the most common metabolic bone disorder affecting more than 10 million individuals in the United States alone [1]. Due to loss of bone mass and compromised bone strength, a significant number of osteoporotic patients experience fragility fractures. Vertebral compression fractures are one of the most common fragility fractures in osteoporotic patients [2], and the incidence of vertebral fracture is steadily increasing with the growing population of older adults.

For osteoporotic patients with a vertebral body fracture, conservative management with bed rest, bracing, and pain management with analgesics remains the mainstay of treatment [3]. When initial conservative management fails, percutaneous vertebral body augmentation procedures such as vertebroplasty or kyphoplasty are commonly undertaken to help relieve pain and disability [4, 5]. However, some patients suffer progressive collapse or nonunion of the vertebral body that results in focal kyphotic deformity and neurologic compromise from retropulsion of bony fragments into the spinal canal [6, 7]. For these patients, percutaneous vertebral body augmentation procedures are contraindicated with concerns relating to aggravation of fragment retropulsion and spinal cord compression. To address these concerns, surgical reconstruction of the anterior spinal column should be considered to restore spinal alignment following anterior decompression. Although rare, complications following percutaneous vertebral augmentation procedures using polymethylmethacrylate (PMMA) cement such as infection, recollapse, and cement dislodgement necessitate surgical treatment for resection of the lesion and reconstruction of the anterior spinal column [8]. However, due to mechanical and biological vulnerability of osteoporotic bone, reconstruction of osteoporotic spinal columns frequently results in subsidence and failure of fixation [9].

Structural femoral shaft allografts are commonly used to reconstruct segmental defects following revision arthroplasty or resection of tumorous or infected bone. Furthermore, femoral shaft allografts are occasionally used for interbody spine fusion or vertebral replacement for patients with tumours, acute fractures, and spinal deformities [10, 11]. Therefore, we hypothesize that femoral shaft allografts could be a viable option for replacement of a collapsed vertebral body in osteoporotic spinal columns. Due to the advantages that femoral shaft allografts have over metal implants in terms of their morphometric and biomechanical properties, they are less likely to experience penetration into and subsidence of the weak vertebral body endplates in osteoporotic spines. The purpose of this study was to investigate the clinical and radiographical outcomes of anterior spinal column reconstruction using femoral shaft allografts for osteoporotic patients with vertebral collapse.

## 2. Materials and Methods

This study was performed according to the guidelines of and with approval from the Institutional Review Board of our institution. From January 2004 to June 2014, 21 consecutive patients who were diagnosed with complicated osteoporotic compression fracture (i.e., with progressive pain, disability, severe kyphosis causing sagittal imbalance, neurologic compromise, or treatment failure after initial conservative treatment or vertebroplasty) and had undergone anterior vertebral corpectomy and replacement of vertebra(e) by structural femoral shaft allografting with anterior or posterior instrumentation were included in the study. The exclusion criteria were (i) pathologic fracture, (ii) traumatic compression or burst fracture, and (iii) use of a cage for vertebral replacement. All patients were observed clinically and radiographically for a minimum of 1 year. Each patient's medical records and radiographs were reviewed for demographics, diagnosis, ambulatory status, level of lesion, operative findings, and information about clinical and radiographic follow-up (Table 1).

**Figure 1:**
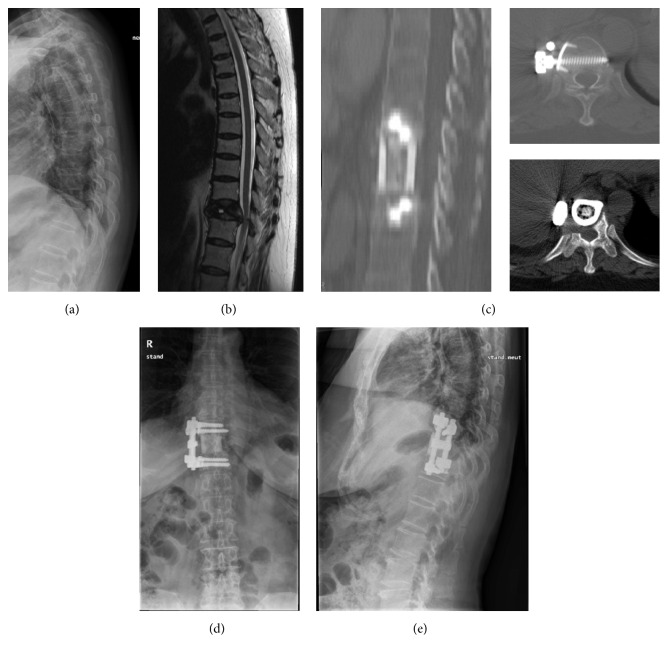
Illustrative case showing the authors' surgical technique of anterior corpectomy with thorough decompression to anterior epidural space and reconstruction with femoral shaft allograft and dual rod and screw construct with staple. Preoperative simple lateral radiograph (a), T2 weighted sagittal MRI (b), postoperative CT with sagittal and axial image (c), 2-year postoperative simple AP (d), and lateral (e) radiography.

**Figure 2:**
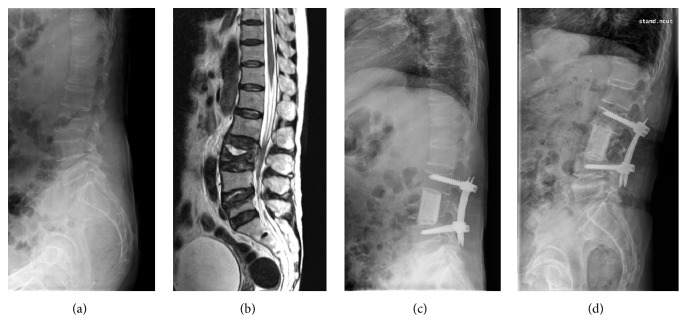
Illustrative case showing the authors' surgical technique of anterior corpectomy and reconstruction with femoral shaft allograft and posterior percutaneous fixation. Preoperative simple lateral radiograph of AP (a), T2 weighted sagittal MRI (b), postoperative simple lateral radiograph immediately after surgery (c), and 2 years after surgery (d).

**Figure 3:**
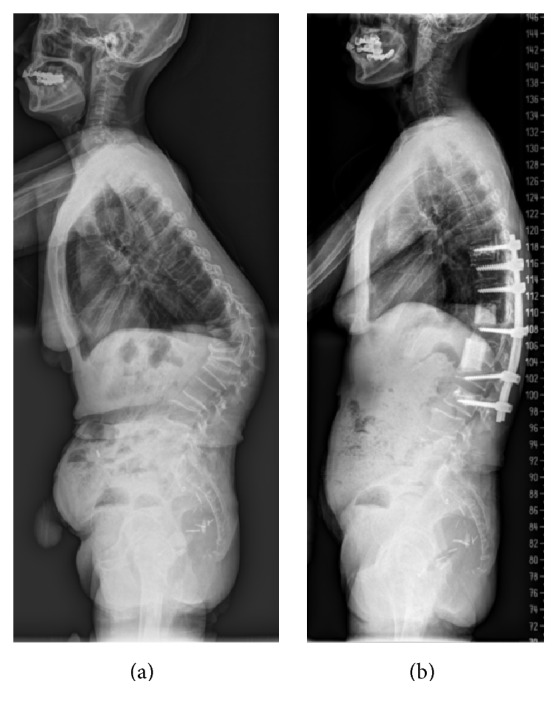
Illustrative case using femoral shaft allograft for correction of global sagittal imbalance that resulted from multilevel osteoporotic compression fractures. Preoperative (a) and 2-year postoperative (b) simple lateral radiograph.

Surgical anterior column reconstruction was performed with freeze-dried, segmental, femoral shaft allografts following corpectomy of the affected vertebra(e) via an anterior transthoracic, retroperitoneal, or combination approach across the diaphragm according to location of the lesion. For both, patients without neurologic compromise and those with neurologic compromise associated with dynamic compression of the spinal cord because of intravertebral instability with nonunion or Kummell's disease, the surgical goal was to achieve rigid reconstruction of the spinal column without direct decompression of the anterior epidural space. For patients with neurologic compromise due to spinal cord compression from retropulsion of bony fragments (usually in cases of a healed burst fracture) or dislodged bone cement, thorough decompression of the anterior epidural space was performed under a microscope prior to grafting and fixation. Anterior instrumentation was performed with Kaneda's technique using staples, screws, and a cross-linked double-rod construct (Figure 1) [12]. Posterior instrumentation was performed with percutaneous or conventional pedicle screws (Figures 2 and 3). Since the femoral shaft only has a solid cortical component surrounding a hollow medullary space without osteoconductive, osteoinductive, or osteogenic potential, autologous bone from resected vertebral body (not in complicated cases with infection of bone cement) or rib was packed with additional fresh frozen allograft bone chips into the middle of the femoral shafts prior to grafting. All patients started ambulation as soon as possible, within 1 week after surgery, and thoracolumbosacral orthosis (TLSO) was applied for 12 weeks postoperatively.

Medical records were reviewed to assess clinical outcomes, perioperative complications, and surgical invasiveness in terms of operation time and estimated blood loss. For assessment of clinical outcomes, ambulatory function was evaluated using a modified Nurick scale: Grade 1, no difficulty in walking; Grade 2, mild effects on gait; Grade 3, difficulty in walking alone or able to walk a few steps with assistance; Grade 4, difficulty standing or able to stand with assistance; Grade 5, chair-bound or bedridden.

*Modified Nurick Grade to Assess Ambulatory Function*

(0) Signs or symptoms of root involvement but without evidence of spinal cord disease
(1) Signs of spinal cord disease but no difficulty in walking
(2) Slight difficulty in walking which does not prevent full-time employment
(3) Difficulty in walking alone; able to walk a few steps with assistance
(4) Barely able to walk; only stands with someone else's help or with the aid of a frame
(5) Chair-bound or bedridden

Severity of subjective pain was measured using the visual analog scale (VAS) score for back pain and/or radiating leg pain, with 0 indicating no pain and 10 indicating severe pain. For radiographic assessment, Cobb's angle of segmental kyphosis, allograft subsidence, subsequent postoperative fractures, and instrument-related complications including screw loosening and implant breakage were evaluated. Radiographs of the spine were taken in the standing position in anteroposterior and lateral views and multiplanar reconstruction was used to examine computed tomography images. Clinical and radiographic results were assessed preoperatively, postoperatively within the 2 weeks before discharge, and at the time of the last follow-up. Observers independent of the main surgeons evaluated clinical and radiographic assessments.

Statistical analysis was performed using SPSS software (version 21.0, SPSS Inc., Chicago, IL, USA). A Wilcoxon rank-sum test was used to for statistical comparisons between preoperative and postoperative, preoperative and final follow-up, and postoperative and final follow-up endpoints. A *p* value ≤0.05 was considered statistically significant. Data are presented as mean (range) or mean (95% confidence interval, CI) unless otherwise stated.

## 3. Results

The mean age of the study subjects, 3 men and 18 women, was 74.0 years (range, 57 to 85 years). Thirteen patients underwent surgery for treatment of delayed collapse of osteoporotic vertebral fractures with spinal cord compression, seven patients for complications following vertebroplasty, and one patient for marked sagittal imbalance related to multilevel kyphotic deformity in the thoracolumbar junction. Levels of affected vertebrae were from T10 to L5. Preoperative mean bone mineral density (BMD) of the lumbar vertebrae (L1 to L4) was 0.614 g/cm^2^ (range, 0.475 to 0.713 g/cm^2^) as determined by dual-energy X-ray absorptiometry (DEXA). Three patients had been treated with osteoporosis medication preoperatively: one with zoledronate and two with alendronate. The mean body mass index (BMI) of patients included in the study was 23.4 kg/m^2^ (range, 15.7 to 29.5 kg/m^2^). The mean follow-up period was 47.6 months (range, 12 to 113 months). Instrumentation was performed anteriorly in 13 patients and posteriorly in 8 patients. Thirteen of 14 patients with lower thoracic to proximal lumbar level disease between T10 and L2 underwent anterior instrumentation (10 single level and 3 double level diseases) (Figure 1). Five patients with lower lumbar level disease from L3 to L5 underwent posterior instrumentation (Figure 2). For three patients with sagittal imbalance requiring multilevel instrumentation, long posterior fixation with pedicle screws and rods was performed (Figure 3). All instrumentation was done on the day of corpectomy with the exception of three patients who underwent multistage operations. Two of three patients with infected vertebroplasty underwent multistage posterior instrumentation after antibiotic treatment following anterior corpectomy. Another patient with iatrogenic flat back syndrome due to a previous lumbar fusion from L2 to L5 complicated with the recollapse of a PMMA augmented L1 vertebra underwent multistage posterior long level surgery with pedicle subtraction osteotomy at L3 followed by anterior corpectomy of L1 (Table 1).

Mean operative time was 223.1 minutes (range, 140 to 360 minutes). Mean estimated blood loss was 721.9 mL (range, 350 to 1800 mL). Before surgery, the median Nurick grade of all patients was 4 (range, 4 to 5) and it significantly improved to 3 (range, 2 to 4) at the final follow-up (*p* < 0.05). All patients could walk with or without a cane at the time of discharge. The mean preoperative VAS score of back and/or leg pain was 6.6 (range, 3 to 9) preoperatively and significantly improved to 3.3 (range, 1 to 5) postoperatively, at the time of discharge (*p* < 0.05).

**Figure 4:**
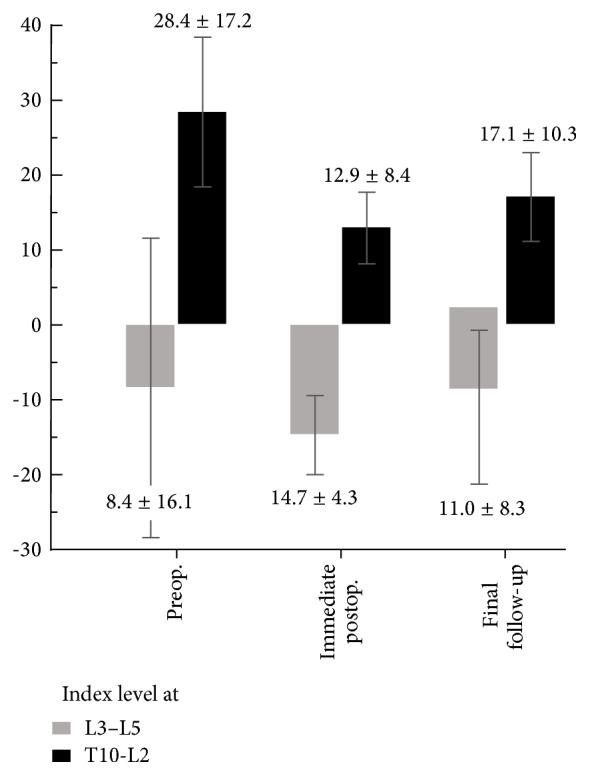
Change of segmental kyphosis or lordosis angle before and after surgery according to the level of the lesion.

The Cobb's angle of segmental kyphosis in the thoracic and thoracolumbar area was improved from 29.1 degrees (95% CI, 20.4 to 37.8 degree) preoperatively to 10.6 degrees (95% CI, 6.4 to 14.7 degree) postoperatively and slightly worsened to 16.1 degrees (95% CI, 10.5 to 21.7 degree) at the final follow-up. The Cobb's angle of segmental lordosis in the lumbar area was 8.4 degrees (95% CI, − 11.5 to 28.4 degree) preoperatively, 14.7 degrees (95% CI, 9.4 to 20.0 degree) postoperatively, and 11.0 degree (95% CI, 0.8 to 21.3 degree) at the final follow-up. The kyphosis angle and lordosis angle immediately after surgery were both significantly improved (*p* < 0.05), and although both the kyphosis and lordosis angles showed slight progression at final follow-up, the differences were not statistically significant. The radiographically derived average subsidence of femoral shaft allografts at final follow-up was 1.86 mm (95% CI, 0.91 to 2.82 mm). There were 2 cases of marked progression of kyphosis (i.e., an increase >15 degrees), which were concluded to be related to the loosening of screws rather than to subsidence or penetration of the graft into the endplates of adjacent vertebra(e). This conclusion was made because the subsidence in each case was only 1.5 mm and 4.2 mm, respectively (case number 10 and case number 16, Table 1). Eleven patients underwent a transthoracic surgical procedure with the chest tube removed at an average of 4.6 days postoperatively. Three patients experienced mild atelectasis within 2 or 3 days postoperatively and were treated with supportive lung care. Discharge was delayed for 2 patients because of pulmonary complications: one due to pneumonia and 1 due to hydropneumothorax. One patient exhibited delayed infection that occurred as psoas abscess in combination with discitis one level below the index level of surgery. This patient underwent surgical debridement and antibiotic treatment without removal of the graft and associated instrumentation. During follow-up, benign compression fracture of the adjacent vertebral body was found in six cases, that is, 38% of the study population. Three of these patients complained of severe pain and were therefore treated with vertebroplasty. The other three patients were treated with TLSO, and no additional surgical intervention was required. In spite of additional fractures in adjacent levels, the femoral shaft allografts were well maintained in 90.5% of all patients (i.e., 19 of 21 cases), with the two aforementioned cases in which the Cobb's angle of segmental kyphosis increased by more than 15 degrees (Figure 4).

## 4. Discussion

**Figure 5:**
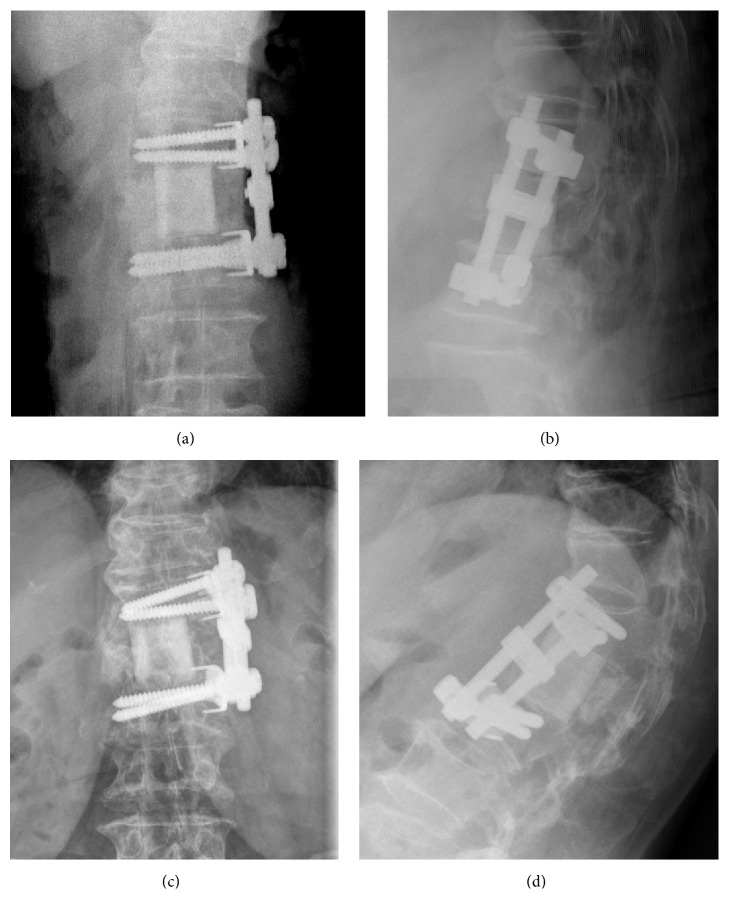
Marked progression of kyphosis of the construct related to loosening of screw fixation and without notable subsidence or penetration of the graft into the adjacent endplate. Preoperative simple radiographs of AP (a) and lateral (b) view and follow-up simple radiograph of AP (c) and lateral view (d) 4 years after surgery.

The results of this study demonstrate that the use of femoral shaft allografts as an anterior column support in between osteoporotic vertebrae results in minimal graft subsidence. The frequent subsidence of grafts or vertebral replacement materials such as mesh cages and the loosening of instruments used for fixation are two of the most common issues that deleteriously affect the efficacy and longevity of constructs used in the reconstruction of osteoporotic spines [13, 14]. Kanayama et al. [15] reported that 20% of patients who underwent anterior spinal reconstruction using ceramic spacers, titanium cages, or iliac bone graft needed additional posterior reinforcement owing to early progression of kyphosis or instrument failure. Anterior column support with a cage of a larger diameter or with multiple cages has been proposed as an approach to avoid high rates of cage subsidence into the adjacent vertebral endplates in osteoporotic patients [16, 17]. However, even in patients with osteoporosis, it is difficult to avoid subsidence with metal implants such as mesh cages. For example, the mean subsidence of titanium mesh cages used for anterior column support in nonosteoporotic patients was reported to be 2.6 mm, with subsidence greater than 5 mm in some cases [18]. The current study using femoral shaft structural allografts for anterior column support showed significant improvement of focal kyphosis after reconstruction surgery. The allograft construct was found to be well maintained until final follow-up with a mean subsidence of 0.8 ± 3.7 mm. Failure of the femoral shaft allograft was observed in only 9.5% of the study population (2 of 21 patients), and it was determined that failure was due to loosening of fixation rather than subsidence of the graft. (Figure 5) Longer posterior instrumentation or combined anterior/posterior fixation is supposed to be considered to prevent that kind of loosening of fixation in addition to PMMA augmented screw-fixation technique [19, 20].

For successful anterior column support, vertebral replacement should provide enough strength to transmit axial compressive forces of the body and/or trunk without penetrating into the adjacent vertebral endplates. Femoral shaft structural allografts exhibit biomechanical properties adequate to support the trunk [21], and, due to their larger contact surface and diameter, interface with the stronger peripheral regions of adjacent endplates thereby decreases the likelihood of subsidence. Another advantage of femoral shaft allografts is that their modulus of elasticity is more similar to autologous bone than to implants made from metal or other artificial materials. As a result, femoral shaft allografts are superior in avoiding stress-shielding effects that reduce bone density and prohibit bone union. Authors suggest that any other type of implant with a larger contact surface and similar mechanical properties allowing load transfer without penetrating into endplate and which also can provide bony integration could be an ideal option for replacement of osteoporotic vertebra(e).

However, the major drawback while using femoral shaft allograft as an anterior spinal column support is the potential for pulmonary complications in elderly patients following anterior thoracotomy. In this study, 45.5% of the patients who underwent anterior thoracotomy (5 of 11) experienced pulmonary complications including 1 case of pneumonia, 1 case of hydropneumothorax, and 3 cases of mild atelectasis. It is for this reason that many recent reports favour posterior approaches using pedicle screws and rod constructs for stabilization and anterior column support in vertebral body augmentation with vertebroplasty or shortening osteotomy [14]. However, some authors still advocate anterior surgery in combination with anterior or posterior instrumentation, emphasizing the importance of rigid anterior column support with bone-to-bone strut grafts and the advantages of a short fusion level [19, 22]. If both posterior and anterior approaches are applicable, authors usually favour the posterior approach with vertebroplasty, especially for patients with collapsed vertebra and neurologic deficit due to intravertebral instability associated with nonunion or pseudarthrosis of osteoporotic vertebral compression fractures. However, there are still patients in whom anterior surgery is favoured in order to provide clearance of the spinal canal and realign the spinal column. For example, anterior surgery is favoured in the case of a fixed fragment compressing the spinal cord or causing severe kyphosis [12, 19, 22]. In these cases, the use of a femoral shaft allograft provides a viable option as an anterior column support if the postoperative pulmonary risk is deemed acceptable. In this vein, modern advances in minimally invasive anterior surgical approaches hold potential to reduce associated pulmonary complications [23].

Although this study was limited in terms of its retrospective design, relatively small number of cases, and lack of controls, we believe that the results presented suggest that femoral shat allografts are a promising alternative approach for anterior column support in osteoporotic spines. Anterior spinal column support using femoral shaft structural allografts in patients with osteoporosis is beneficial in terms of avoiding subsidence and maintaining the mechanical integrity of the construct. However, additional clinical experience and studies including larger patient populations with long-term follow-up are necessary to validate the advantages of femoral shaft allografts.

## 5. Conclusion

This study demonstrated that the use of femoral shaft allografts as an anterior column support in between osteoporotic vertebrae improved kyphosis and resulted in minimal subsidence. We concluded femoral shaft allografts as an effective treatment option for dealing with osteoporotic vertebral collapse and kyphotic deformity.

## Figures and Tables

**Table 1:** Details of 21 study patients.

Case number	Age	Sex	Diagnosis	Ambulatory status	Level of lesion	Instrumentation	Follow-up (months)
1	72	F	Delayed collapse after fracture with spinal cord compression	Assisted standing	T12, L1	Anterior dual rod and screw with staple	113
2	85	F	Delayed collapse after fracture with spinal cord compression	Assisted standing	T12	Anterior dual rod and screw with staple	81
3	68	M	Delayed collapse after fracture with spinal cord compression	Assisted standing	L5	Posterior conventional pedicle screw	14
4	76	F	Recollapse after vertebroplasty with dislodgement of PMMA, with spinal cord compression	Assisted standing	T12, L1	Anterior dual rod and screw with staple	84
5	72	F	Recollapse after vertebroplasty with dislodgement of PMMA, with spinal cord compression	Assisted standing	T12	Anterior dual rod and screw with staple	54
6	79	F	Delayed collapse after fracture with spinal cord compression	Assisted standing	L3	Posterior percutaneous pedicle screw	83
7	61	F	Delayed collapse after fracture with spinal cord compression	Assisted gait	T10	Anterior dual rod and screw with staple	80
8	60	F	Delayed collapse after fracture with spinal cord compression	Assisted gait	T11, L1	Anterior dual rod and screw with staple	64
9	83	F	Infected vertebroplasty with spinal cord compression	Impossible	L1	Anterior dual rod and screw with staple	60
10	70	F	Recollapse after vertebroplasty with spinal cord compression	Assisted standing	L1	Anterior dual rod and screw with staple	52
11	77	F	Delayed collapse after fracture with spinal cord compression	Assisted standing	L4	Posterior percutaneous pedicle screw	36
12	78	F	Delayed collapse after fracture with spinal cord compression	Impossible	L1	Anterior dual rod and screw with staple	18
13	74	F	Delayed collapse after fracture with spinal cord compression	Assisted standing	T12	Anterior dual rod and screw with staple	12
14	84	F	Delayed collapse after fracture with spinal cord compression	Assisted standing	L1	Anterior dual rod and screw with staple	14
15	78	F	Delayed collapse after fracture with spinal cord compression	Impossible	L4	Posterior percutaneous pedicle screw	23
16	79	F	Delayed collapse after fracture with spinal cord compression	Assisted standing	L2	Anterior dual rod and screw with staple	20
17	57	F	Multilevel kyphotic collapse with marked sagittal imbalance	Assisted gait	T11, L1	Posterior conventional pedicle screw	18
18	79	M	Infected vertebroplasty with spinal cord compression	Impossible	L1	Posterior conventional pedicle screw	12
19	77	F	Infected vertebroplasty with spinal cord compression	Impossible	L4	Posterior percutaneous pedicle screw	13
20	70	M	Delayed collapse after fracture with spinal cord compression	Impossible	L2	Anterior dual rod and screw with staple	13
21	75	F	Recollapse after vertebroplasty on postoperative flatback	Impossible	L1	Posterior conventional pedicle screw	12
